# Thinking Outside the Euclidean Box: Riemannian Geometry and Inter-Temporal Decision-Making

**DOI:** 10.1371/journal.pone.0145159

**Published:** 2016-03-28

**Authors:** Himanshu Mishra, Arul Mishra

**Affiliations:** 1 David Eccles School of Business, University of Utah, Salt Lake City, Utah, United States of America; 2 1655 E. Campus Center Drive, SFEBB 7111, David Eccles School of Business, University of Utah, Salt Lake City, UT 84112, United States of America; University of Sussex, UNITED KINGDOM

## Abstract

Inter-temporal decisions involves assigning values to various payoffs occurring at different temporal distances. Past research has used different approaches to study these decisions made by humans and animals. For instance, considering that people discount future payoffs at a constant rate (e.g., exponential discounting) or at variable rate (e.g., hyperbolic discounting). In this research, we question the widely assumed, but seldom questioned, notion across many of the existing approaches that the decision space, where the decision-maker perceives time and monetary payoffs, is a Euclidean space. By relaxing the rigid assumption of Euclidean space, we propose that the decision space is a more flexible Riemannian space of Constant Negative Curvature. We test our proposal by deriving a discount function, which uses the distance in the Negative Curvature space instead of Euclidean temporal distance. The distance function includes both perceived values of time as well as money, unlike past work which has considered just time. By doing so we are able to explain many of the empirical findings in inter-temporal decision-making literature. We provide converging evidence for our proposal by estimating the curvature of the decision space utilizing manifold learning algorithm and showing that the characteristics (i.e., metric properties) of the decision space resembles those of the Negative Curvature space rather than the Euclidean space. We conclude by presenting new theoretical predictions derived from our proposal and implications for how non-normative behavior is defined.

## Introduction

Inter-temporal decisions involve deciding in favor of an outcome occurring in the present (e.g. receiving $200 now) versus that occurring in the future (e.g. receiving $300 after 6 months). Inter-temporal decisions are common among humans and animals and affect many decisions such as whether to have a health check-up right now or delay and face the consequences, save versus spend now, consume indulgent food now and face the consequences later, hoard food now or wait till later. Across both human and animal studies, it has generally been found that organisms i.e. the decision-makers, are likely to overweigh immediate, but smaller rewards over delayed and larger rewards. These trade-off decisions significantly influence impulse control, addictive behavior, retirement savings, investment, procrastination, attitude towards climate change, etc. [1–7]. Therefore, understanding how humans and animals make inter-temporal decisions is a much-researched area having implications in myriad domains.

Many computational models have been presented to explain inter-temporal decisions. These models use discount functions, which are decay/depreciation functions (see [8]) where the current monetary or non-monetary reward decays as its receipt is pushed into the future. The first formal discount function came from the Discounted Utility (DU) model by Samuelson [9]. The DU model, which proposes a constant rate discount function (e.g. exponential discounting), was presented as the normative model suggesting how decision-makers should behave when presented with inter-temporal decisions [10]. Constant rate discounting works as follows: the utility of $*x* decays at a constant rate per unit of time. $*x* today would be worth $*xδ* after one year, $*xδ*^2^ after 2 years and so on. Here *δ* is <1 and remains constant over the time horizon. As an alternate to the DU model, variable rate discount models have also been proposed to explain empirical evidence in inter-temporal decision-making. Variable rate discount models, (e.g., hyperbolic discounting models) [11, 12], suggest that people’s discount rate decreases over time i.e. it is higher in proximate time intervals but lower in distant intervals. One simple way to understand differences between constant rate and variable rate discount models is to compare them to a bucket of water with a hole that leaks water as one pushes it along a straight path. If we replace the water with money and the straight line with the temporal line then according to constant rate models, for each unit of temporal distance covered, the leakage (depreciation) is at a constant rate. In this manuscript, for ease of description, we use money as a reward or source of utility. Money can be replaced with any other source of utility. For variable rate models, the leakage is a function of temporal distance covered. Another way to see the difference between constant rate and variable rate models is to consider their corresponding differential equations. If we assume that at time *t* = 0 the initial monetary value is $*y* and *y*(*t*) represents the residual value at time *t* then constant rate models can be conceptualized as a solution to linear autonomous first order differential equation of the form: $\frac{\frac{dy}{dt}}{y}=-\psi$ where *ψ* is the constant discount rate. On the other hand, variable rate models can be conceptualized as a solution to the linear nonautonomous first order differential equation of the form: $\frac{\frac{dy}{dt}}{y}=-\Omega \left(t\right)$ where the discount rate is a function of time. At a broader level this captures the main difference between these two types of models.

As with any line of research, questions have been raised about both the constant rate and the variable rate models, including questions about their ability to explain empirical evidence, their theoretical underpinnings and their predictive/explanatory power [13]. Moreover recent research has suggested that recognizing the way we perceive time may not be the same as objective time, could help us better understand inter-temporal decisions. Specifically, some distinctions between constant and variable rate models fade if we formulate time perception to be non-linear (e.g., logarithmic) [14, 15].

Before we describe some commonalities between various discount models, we first need to explain a term that we will be using frequently to discuss our proposed model i.e., “decision space”? One of the objectives of research involving any type of decision-making is to understand how the organism or the decision-maker perceives a decision-object (e.g. given information). Such a perception forms her subjective experience of decision objects and the decision hinges on this perception. We refer to the space where information is perceived and the decision is made as the decision space or decision surface of that decision-maker. If we take the context of inter-temporal decision-making, then the subjective perception of time and reward/money occurs in the inter-temporal decision space of the decision-maker.

Despite their much-discussed differences, there are some important commonalities among the various discount models. First, most existing models assume the inter-temporal decision space to be a metric space. For instance, a decision of comparing the receipt of $100 now to receiving $250 at a point in time eighteen months in the future has been studied using a discount function of temporal distance estimate (e.g., *δ*(*t*) where *t* is the distance between these 2 points in time). The assumption of a metric space implies that by using a metric we can estimate the length of a time interval used in decision-making. Specifically, if *t*_1_ < *t*_2_ < *t*_3_ are three points on a temporal line then inter-temporal decision space being metric space means that (i) the distance between *t*_1_ and *t*_1_, *d*(*t*_1_ → *t*_1_) = 0, (ii) *d*(*t*_1_ → *t*_2_)>0, (iii) *d*(*t*_1_ → *t*_2_) = *d*(*t*_2_ → *t*_1_) and (iv) *d*(*t*_1_ → *t*_2_)+*d*(*t*_2_ → *t*_3_)≥*d*(*t*_1_ → *t*_3_). The important aspect of considering the inter-temporal space to be a metric space is that we need to know what is the geometry of the surface on which the distance is being measured because any accurate distance estimate is entirely dependent on the surface on which the distance is being measured and is defined by the unique metric of that surface. Therefore, by thinking of temporal comparisons in terms of distance (which almost all the previous research does), a discussion of the geometry of the surface on which temporal distance is being measured is unavoidable.

This brings in the second point on which all existing models of inter-temporal decision-making converge. Existing models assume the metric of the inter-temporal decision space to be a Euclidean metric since the temporal distance between *t*_1_ and *t*_2_ (where *t*_2_ > *t*_1_) is calculated as: $d\left(t_{1}\to t_{2}\right)=t_{2}-t_{1}=\sqrt{{\left(t_{2}-t_{1}\right)}^{2}}$, which is similar to measuring the Euclidean distance between points (*t*_1_, 0) and (*t*_2_, 0). The decision space is assumed to be a metric space with an underlying Euclidean geometry.

Therefore, an important similarity across all existing models of inter-temporal decision-making is the assumption of a Euclidean decision space. However, despite such a fundamental assumption of a Euclidean space, to the best of our knowledge, no research has ever questioned this assumption and neither has anyone empirically tested for whether the assumption is true or not. Let us explain next why this might be so.

Such an unquestioning assumption of a Euclidean space is not surprising. Due to its intuitive appeal Euclidean geometry has always had a pervasive influence on scientific inquiry. The best example of this influence can be seen in Kant’s ([16, 17]: originally published in 1781) arguments suggesting that Euclidean space is a-priori intuition in human judgments. It matches with how the world appears visually to us and is incredibly accurate in many everyday predictions. Given the intuitive appeal and face-validity of Euclidean space, it is not surprising to see that in the fields that explore human preferences, whenever metric space needed to be defined, either we explicitly assumed the metric space to be Euclidean ([18] pg 417, [19] pg 674) or implicitly used it to model behavior without further testing for its veracity empirically. While Kant’s argument could be accepted before the introduction of non-Euclidean geometries, it seems less defensible now to assume without empirical investigation that the geometric space underlying inter-temporal decision can only be Euclidean. In keeping with [20] and [21], who were among the first to consider the notion that the geometry of space around us is an empirical matter that should be decided by measurements, we need to ask why should we assume that time and rewards are perceived by the decision-maker in a Euclidean space? What is the basis for such an assumption? Does empirical evidence support such an assumption? Might there be a different geometry involved when time and rewards are perceived by the decision maker?

First, we need to ask whether our conclusions, predictions, and explanations would change if we considered a Euclidean geometry when the geometry was actually non-Euclidean? The straightforward answer is that if the space is curved and we assume it to be flat, we will introduce distortions in measuring the shortest distance (called the geodesic distance). It is analogous to assuming that the earth is flat and trying to measure distance between two points. However, once we know that the surface of the earth is spherical then we can use the correct metric to estimate distance. Similarly, if we considered the wrong geometry for the inter-temporal decision space, we would be introducing distortions in our measurements and subsequently in how we assess the discounting process. In such a situation infinite distance functions can be proposed and some of them would work some of the times. This is the current situation in inter-temporal distance estimates. Therefore, determining the correct geometry of the decision space is the essential first step needed to apply the appropriate metric to calculate distances on that space and as a consequence our distance estimates would be distortion-free. Second, it would help us build a theoretical model that would better explain the empirical findings and help make further theoretical predictions.

## Overview

How does a decision-maker evaluate the utility from different bundles of time and corresponding monetary pay-offs for that time? He does so by perceiving those bundles at different distances in his decision space. To compare different bundles he brings each bundle to the present (origin) and calculates the discounted utility from each bundle. The discounting is a function of the perceived distance. Therefore, in this research our attempt is to find out the correct geometry of the decision space to ensure that our distance estimates are accurate.

Previewing briefly, in this research, we present a new model of inter-temporal decision-making by relaxing the current restrictive assumption that the decision space is a Euclidean space. We develop our theory on the proposal that the decision space resembles the metric properties of a more flexible Riemannian space of constant negative Gaussian curvature. This implies that the metric used by the decision maker is not Euclidean but the more general Riemannian metric. We further suggest that both temporal distance and reward influence the magnitude of discounting. We discuss this proposal in detail in a later section (The Proposed Model). In order to maintain consistency, we use the term Riemannian space throughout the manuscript. Another term that can be used is Riemannian manifold. In questioning the Euclidean nature of the decision space we follow previous research in other domains that have considered non-Euclidean spaces. Apart from the most well known use of non-Euclidean space in the general theory of relativity [22], researchers in many domains, such as Embedding of networks [23], binocular vision and perception [24–26] to name a few have questioned the Euclidean assumption and searched for solutions using non-Euclidean spaces.

Two arguments, interestingly opposing, can be raised against our research. One that inter-temporal decision-making is a relatively smaller problem that doesn’t require the mathematical complexity of curved spaces. We argue that this may be true if we narrowly define the domain of inter-temporal decisions. However, if we consider the concept of a decision space, which plays a critical role in almost every decision we make, this approach has implications for many types of decisions. Conversely, the second argument could question the need for replacing the current Euclidean assumption, which makes it easy to understand and apply existing discounting models, with an elaborate process of questioning the geometry of the decision space. Again, we argue that it is not appropriate for us as researchers to keep assuming something because it is easy to understand and not question it or test for it empirically. For a quick reference to symbols used in this manuscript, please refer to S1 Text.

The remaining part of the manuscript is structured in the following manner. First, in order to build our proposition that a Riemannian space of constant negative curvature underlies the decision space, we provide a brief overview of Riemannian space and Gaussian curvature and explain Riemannian spaces of constant negative curvature (we will refer to it as Negative Curvature spaces or negatively curved spaces). Our discussion of these topics is nowhere close to being exhaustive. These topics are active areas of research across many disciplines. For more in-depth understanding, please refer to the following sources: [27–34]. For a lucid overview see [35, 36]. For a discussion of non-Euclidean geometry using real projective geometry see [37]. (For a detailed explanation of their mathematical roots please refer to S2 Text). Second, we present our proposed geometric theory of inter-temporal decision making. We explain how information is perceived in the decision space. Third, we use two approaches, analytical and empirical, to test our proposed theory. We test it analytically by examining whether we can explain the existing findings in inter-temporal decision making when we utilize a discount function that uses distance in the negatively curved space instead of the Euclidean temporal distance as used in past work. Empirically, we estimate the curvature of the decision space, utilizing inter-temporal decisions made by participants, to test whether it is Euclidean or Negative Curvature space. Finally, we conclude with further theoretical predictions that can be derived from the proposed geometric theory.

## Geometries and Distances

Riemann [20] proposed that spaces do not have any inherent geometry instead they are akin to a continuum where points are specified by their coordinates. Whenever a specific metric is used to measure the distance between two points, it means that an assumption has been made about the geometry of the surface since a metric is unique to a geometry. The problem is that different metrics can be used to estimate the distance and hence, different geometries can be imposed. However, which is the correct metric can be verified only when one knows what is the geometry of the surface on which the points are located. Let’s refer back to the example of a ruler (with the familiar Euclidean metric $ds=\sqrt{dx_{1}^{2}+dx_{2}^{2}+..dx_{n}^{2}}$) versus a cooked spaghetti. If asked to measure the distance between any two points, we can use the ruler or the spaghetti as our measure. If we used the ruler we will get “a” distance estimate, irrespective of whether the surface is flat or curved. The estimate with the ruler would be correct for the flat surface. However, if the surface were curved then the distance estimate using the ruler would be distorted because the ruler is not faithfully representing each point in its distance estimate. The fact that we are getting “an” estimate does not mean that we have the “right” estimate. But how can we know that we have the right estimate? It is possible only if we knew the geometry of the surface on which the points are located. We can attest to the veracity of our estimate only if we empirically test for the geometry of the surface. Hence, [21] had proposed that assumed geometry should always be empirically verified.

In order to identify the right geometry we should have the ability to distinguish between infinite geometries that are possible between points. Such ability would allow us to decide, for instance, whether the right metric is a ruler or a spaghetti. The Gaussian curvature proves to be a very useful measure for such differentiation since it differentiates curved surfaces from flat surfaces (please refer to Text A in S2 Text). The Gaussian curvature (*K*) informs us how curved a specific surface is with respect to a flat surface, at a given point. The magnitude of (*K*) tells us how much the surface is bending. If the Gaussian curvature of a surface is the same at every point then we have a constant curvature geometry. An example is a globe on which, the Gaussian curvature is the same at each point. On the other hand, if we consider a crumpled paper, each point on it has a different Gaussian curvature. Pertinent to our research, we will be considering constant curvature geometries. Moreover, the sign, positive or negative, of the Gaussian curvature informs us of the type of geometry in a particular point *p*’s neighborhood. Specifically, at every point on a surface if *K* = 0 then the neighborhood of *p* would resemble a flat surface and its intrinsic geometry would be Euclidean. If *K* > 0 then it would resemble a surface like a piece of sphere and its intrinsic geometry would be Elliptical. If *K* < 0 then it would resemble a saddle and its intrinsic geometry would be Gauss-Bolyai-Lobachevskian/Hyperbolic. Since *K* can have any value between −∞ and ∞, we can think about constant curvature geometries not as three separate geometries, but instead as a continuum of infinite geometries where the Euclidean geometry is a special case. In sum, the Gaussian curvature of the space helps distinguish among various geometries.

The geometry we assume for a surface will dictate our choice of metric since each geometry is defined by its unique metric. Therefore, a ruler ideal for measuring distances on surfaces with *K* = 0 would not be right to use on a surface with *K* greater than zero. We now discuss the model we use to calculate geodesic (the shortest path between two points) on the Negative Curvature space (please refer to Text B in S2 Text). Since the Negative Curvature space is abstract and hence, difficult to imagine, models are constructed that make it easier to measure distances between points in such abstract spaces. These models tend to be Euclidean representations that faithfully embody the key features of the non-Euclidean geometry. Researchers depend on various models of Negative Curvature space such as Poincarè half space, Poincarè disk, and Beltrami-Klein model to understand the properties of the space. In our proposal we use the hyperboloid model.

To understand the problem of assuming the wrong geometry, let’s compare the Euclidean geodesic with the geodesic on a negatively curved space between two points p and q. We use the geodesic (shortest distance) estimates for Euclidean distance and the distance on a hyperboloid. If **p** = (*p*_1_, *p*_2_, *p*_3_, … .*p*_*n*_) and **q** = (*q*_1_, *q*_2_, *q*_3_, … .*q*_*n*_) are two points in *E*^*n*^ (*n*-dimensional Euclidean space) then their geodesic distance in the Euclidean space is

$$\begin{array}{ll} d_{e}\left(p,q\right) & =\sqrt{{\left(q_{1}-p_{1}\right)}^{2}+{\left(q_{2}-p_{2}\right)}^{2}+\cdots +{\left(q_{n}-p_{n}\right)}^{2}}\\ & =\sqrt{\sum_{i=1}^{n}{\left(q_{i}-p_{i}\right)}^{2}} \end{array}$$ (1)

However, if these points are on a hyperboloid *H*^*n*^ ⊂ *E*^*n*, 1^ where *K* = −1 then

$$\begin{array}{ll} d_{h}\left(p,q\right) & =Cosh^{-1}\left(-\langle p,q\rangle \right)\\ & =Cosh^{-1}\left(\sqrt{1+\sum_{i=1}^{n}p_{i}^{2}}\sqrt{1+\sum_{i=1}^{n}q_{i}^{2}}-\sum_{i=1}^{n}q_{i}p_{i}\right) \end{array}$$ (2)

For any other value of *K* < 0

$$\begin{array}{c} d_{h}\left(p,q\right)=\frac{Cosh^{-1}\left(K<p,q>\right)}{\sqrt{-K}} \end{array}$$ (3)

As these equations highlight, assuming the wrong geometry introduces distortions in estimates of distance. Please see Text A in S3 Text for the detailed derivation of Eqs 2 and 3.

## The Proposed Model

In this section, we first provide details of the proposed model and then derive various mathematical relationships. We propose that the given information about time and money is perceived in (or mapped on to) the Negative Curvature decision space. Specifically, the following points capture the essence of our proposal.

1. We suggest that the decision space where the perception of information and the subsequent decision takes place resembles the metric properties of Negative Curvature space. The proposal that the decision space is a Negative Curvature space in no way implies that the decision space appears like a hyperboloid embedded in a higher dimension pseudo-Euclidean space to a decision maker. Instead, what it means is that the metric properties of the inter-temporal decision space, as perceived by the decision-maker, resemble the metric properties of the Negative Curvature space. It is important to note that our proposal does not require any new set of assumptions pertaining to the inter-temporal decision space being considered a metric space. As we discussed earlier, existing research already assumes the inter-temporal decision space to be a metric space and uses the Euclidean metric to assess inter-temporal distance. Since the Euclidean space is a special case of Riemannian spaces of constant curvature, we do not need to introduce any new assumptions. Our proposal actually relaxes a very restrictive assumption of zero Gaussian curvature, which is required for a space to be a Euclidean space.
2. We further propose that the Gaussian curvature of the decision space is malleable. Although the decision space would remain one with a negative curvature, the value of the Gaussian curvature could change depending on various factors. We argue consistent with the view espoused by Riemann [see [38] pg 98] that “the space in itself is nothing more than a three dimensional manifold devoid of all form; it acquires a definite form only through the advent of the material content filling it and determining its metric relations”. We propose that dispositional factors of the decision-maker, as well as contextual factors in which the decision is being made, have the ability to change the Gaussian curvature of the decision space and thus, in turn the metric relationship among objects perceived in it.
3. In our proposed model we consider the influence of both time and money on discounting. Therefore, instead of using time as the sole influencer on the discounting process, we use the distance in the time-money decision space. Specifically, when a decision maker is considering two bundles of time and money, (*m*_1_, *t*_1_) and (*m*_2_, *t*_1_), she is doing so from the vantage point of her subjective origin. The best way she can compare the two bundles is by bringing the bundles, by discounting their value appropriately, to the origin. We propose that the discounting factor she uses is the distance *d*_*h*_ of a specific bundle from the point on which it is situated in her inter-temporal decision space to the origin. The difference between our method and previous ones is that previous methods use just time in the discounting factor e.g. $m_{1}\delta^{t_{1}}$ or $\frac{m_{1}}{1+\gamma t_{1}}$. In our model the discounted utility of the (*m*, *t*) bundle is not due to the Euclidean distance between the origin and just *t* but due to the distance between the origin and (*ϕ*(*m*), *η*(*t*)) in the Riemannian space, where *ϕ*(*m*) and *η*(*t*) are the perceived values of money and time respectively.

As we demonstrate later, our model explains many of the existing findings in inter-temporal literature such as those findings, which have been labeled as anomalies as well as those that deal with time perception (e.g. the thesis that time is perceived logarithmically). Hence, our proposal is more inclusive and general. Moreover, by presenting a positive theory of inter-temporal decision-making, we not only explain the existing findings in the literature, we also present predictions (section) based on the theory that can be empirically tested. Although one could argue that we are relaxing geometric assumptions of the inter-temporal framework, we are not adding any additional variables to the framework. Hence, our model is as parsimonious as the others proposed previously.

As an aside, one could question why we are proposing the decision space to have a negative curvature i.e., *K* < 0 ? Why not a positive curvature space with *K* > 0 ? *K* > 0 spaces are elliptical spaces. A good example of *K* > 0 space is a sphere/ball. Let’s imagine that we want to measure the distance between two points A (which is fixed) and B (which is moving) on a ball. As B moves away, we first see that the distance between A and B increases. However, later as B gets farther, its distance from A reduces; eventually B coincides with A and thus, the distance becomes 0 between A and B. This happens because a ball (like elliptical spaces) is a closed surface. If we consider the decision space to have *K* > 0, we will face some logical hurdles. For example, this would imply that as an event moves away from the present time, it’s perceived temporal distance first increases and then decreases. In other words, this would require us to think of time as circular where after a finite delay, the future coincides with the present. It is a notion that is hard to justify logically as well as experientially.

In the next section we derive various mathematical relationships to understand how information is perceived in the inter-temporal decision space.

### Distance Perception in the Decision Space

We use the distance estimate on a hyperboloid to formulate the distance estimate on the inter-temporal decision space. Let’s consider a decision-maker who is contemplating an inter-temporal choice problem from his vantage point at the origin (i.e the present). The decision-maker is asked to make a choice between receiving $*m*_1_ after *t*_1_ delay, versus receiving $*m*_2_ after *t*_2_ delay (where *m*_2_ > *m*_1_ and *t*_2_ > *t*_1_). To decide, he has to compute a discounted value of *m*_1_ and *m*_2_. Let *ϕ*(*m*) denote the value (utility) one perceives in any given amount of money (here *ϕ*(.) is a monotonically increasing function). According to existing literature, if we assume *ϕ*(*m*) = *m*, we would predict that the decision-maker would choose *m*_2_ after *t*_2_ delay if $m_{1}\delta^{t_{1}}<m_{2}\delta^{t_{2}}$ (exponential discounting) or $\frac{m_{1}}{1+\gamma t_{1}}<\frac{m_{2}}{1+\gamma t_{2}}$ (hyperbolic discounting). *δ* < 1 and *γ* is the discounting parameter. We acknowledge that there are various discount functions. The use of exponential and hyperbolic is just to give an example.

In our proposal, we suggest that the decision maker would choose *m*_1_ if *ϕ*(*m*_1_)*f*(*d*_*h*1_) < *ϕ*(*m*_2_)*f*(*d*_*h*2_). This implies that discounting is not a function of *t* (temporal distance) instead it is a function of *d*_*h*_; the perceived distance between the origin and any money-time bundle in the Negative Curvature decision space. *η*(*t*) is a monotonically increasing function of *t* and denotes the magnitude one perceives of any given time interval. Our proposed model can be described as a two-stage model just like any standard discounting model. In the first stage both *ϕ*(*m*) and *η*(*t*) are used to assess distance from the origin. While in the second stage that distance is used to discount the values of *ϕ*(*m*). The following Eq (3), for a particular time/money bundle ($*m* at time *t*), *d*_*h*_ represents the distance of that bundle from the origin (status quo) in the inter-temporal decision space according to

$$\begin{array}{c} d_{h}=\frac{Cosh^{-1}\left(\sqrt{-K}\sqrt{\phi^{2}\left(m\right)+\eta^{2}\left(t\right)-\frac{1}{K}}\right)}{\sqrt{-K}},\;K<0 \end{array}$$ (4)

Please see Text B in S3 Text for a detailed derivation of Eq 4.

In order to make our exposition simpler we adopted the following steps. First, we assumed the Gaussian curvature *K* to be −1 (however, we can certainly generalize this for any value of *K* ∈ (−∞, 0)). Second, in order to present the equations in the manuscript concisely we specify Γ (gamma) to be as follows $\Gamma =\sqrt{1+\phi^{2}\left(m\right)+\eta^{2}\left(t\right)}$. Substituting *K* = −1 in Eq (4), we can write the simplified version of it as

$$\begin{array}{c} d_{h}=Cosh^{-1}\left(\Gamma \right) \end{array}$$ (5)

Please see Text C in S3 Text for a verification of Eq (5) using a different approach. Next we will describe some properties of *d*_*h*_ that will be used at different points in the manuscript. In the subsequent subsection, we derive the discount function.

Inferring from Eq (5), the following are some relevant properties of *d*_*h*_

$$\begin{array}{c} \frac{\partial d_{h}}{\partial t}=\frac{\eta^{\prime }\left(t\right)}{\Gamma \sqrt{\Gamma^{2}-1}} \end{array}$$ (6)

$$\begin{array}{c} \frac{\partial d_{h}}{\partial m}=\frac{\phi^{\prime }\left(m\right)}{\Gamma \sqrt{\Gamma^{2}-1}} \end{array}$$ (7)

Since *ϕ*(*m*) and *η*(*t*) are monotonic functions of *m* and *t* respectively:

$$\begin{array}{c} \underset{t\to \infty }{\text{lim}}\frac{\partial d_{h}}{\partial t}=0 \end{array}$$ (8)

This means that for a constant *m*, as *t* approaches infinity, the rate of change of *d*_*h*_ approaches zero. In other words, *d*_*h*_ remains largely unchanged for increasing values of *t*.

$$\begin{array}{c} \underset{m\to \infty }{\text{lim}}\frac{\partial d_{h}}{\partial m}=0 \end{array}$$ (9)

Similar to the interpretation of Eqs (8) and (9) shows that for any constant value of *t*, *d*_*h*_ remains largely unchanged for higher values of *m*. While we can increase *m*, its perceived magnitude remains unchanged in the Negative Curvature decision space.

$$\begin{array}{c} \frac{\partial^{2}d_{h}}{\partial t^{2}}<0 \end{array}$$ (10)

Eq (10) simply shows that *d*_*h*_ is a concave function with respect to *t*.

We next apply *d*_*h*_, the distance estimate in the Negative Curvature decision space, to inter-temporal decision situations and derive the discount function. We begin by discussing the leaking bucket analogy.

### Leaking bucket and Discount Function

Let’s revisit the Leaking bucket analogy introduced earlier. If a bucket leaks water only when we walk, then inter-temporal decision-making is analogous to predicting how much water will be left in a leaking bucket after walking a certain distance. That is, what would be the discounted values of money after a delay. The approach in existing research has been to assume that the surface on which we walk is a Euclidean surface with time being the only dimension and all the functions that have been developed to explain the loss of water are associated to the variable/constant size of the hole in the bucket.

We suggest that one way to account for the loss of water is to consider the surface we walk on to have a constant negative Gaussian curvature, instead of the more restrictive assumption of zero Gaussian curvature (i.e, a flat Euclidean surface). We further propose that the size of the hole is constant (i.e., the same amount of water leaks for every unit of distance travelled). Hence, we can assume the water in the bucket to be money and let *y*_0_ be the initial amount of money in the bucket and *ψ* be the fraction of money lost per unit of distance travelled on the Negative Curvature surface (note, that we are conceptualizing time to be a continuous variable, however, one would arrive at similar conclusions by treating time as a discrete variable. The only modification in that instance would be the use of first order difference equations rather than differential equations). Formally, this can be written in the form of a differential equation as

$$\begin{array}{c} \frac{d\phi (y)}{dd_{h}}=-\psi \phi \left(y\right) \end{array}$$ (11)

$$\begin{array}{c} \Rightarrow \phi \left(y_{d_{h}}\right)=\phi \left(y_{0}\right)e^{-\psi d_{h}} \end{array}$$ (12)

Please see Text D in S3 Text for a complete solution of Eq 11. One way to understand *ϕ*(*y*_*d*_*h*__) and *ϕ*(*y*_0_) in Eq (12) is to think of the former as the discounted value of the latter. If we substitute *d*_*h*_ with time *t* in Eq (12) and assume *ϕ*(*y*) = *y* (i.e., linear function), we get the exponential discounting function used in the DU models [5]. Now if we assume *ψ* = 1 (unit loss/constant unit discounting) and substitute the value of *d*_*h*_ from Eq (5), we get

$$\begin{array}{c} \phi \left(y_{d_{h}}\right)=\phi \left(y_{0}\right)e^{-Cosh^{-1}\left(\Gamma \right)} \end{array}$$ (13)

More generally, if the Gaussian curvature of the surface is *K* < 0 then

$$\begin{array}{c} \phi \left(y_{d_{h}}\right)=\phi \left(y_{0}\right)e^{\frac{-Cosh^{-1}\left(\sqrt{-K}\sqrt{\phi^{2}\left(y_{0}\right)+\eta^{2}\left(t\right)-\frac{1}{K}}\right)}{\sqrt{-K}}} \end{array}$$ (14)

To maintain consistency we will use Eq (13) quite frequently in the remaining portion of the manuscript. An easy way to understand what distance means in this formulation is to keep either *m* or *t* constant. When *m* is kept constant then distance indicates the perceived magnitude of time. Similarly, if *t* is kept constant then distance shows the perceived magnitude of money.

Next, we provide support for the notion that the decision space is Negative Curvature space through two different methods. First, if our proposal is true then using Eq (13), we should be able to explain the existing inter-temporal findings documented in literature [39, 40]. Second, empirically we should find that the metric properties of the decision space resemble the metric properties of the Negative Curvature space more than those of the Euclidean space.

## Explaining Inter-temporal Findings

Some of the findings that we discuss are inconsistent with only the DU (constant rate discount) model and some with both the DU and the hyperbolic discounting (variable rate discount) model.

### Common Difference Effect

If (*x*, *t*) denotes receiving $*x* at time *t* then the stationarity property of DU model requires that when (*x*_1_, *t*_1_)≻(*x*_2_, *t*_2_) where *x*_2_ > *x*_1_ and *t*_2_ > *t*_1_ then (*x*_1_, *t*_1_ + *n*)≻(*x*_2_, *t*_2_ + *n*) for *n* > 0 also known as constant willingness to wait [15]. However, empirical evidence suggests that (*x*_1_, *t*_1_ + *n*)≺(*x*_2_, *t*_2_ + *n*) (please see [41] for a description of this effect). In other words, violation of stationarity property leads to a preference reversal such that a smaller, sooner reward is preferred over a larger, later reward in the near-future but a larger, later reward is preferred over a smaller, sooner reward in the distant-future (see [12, 42]). It has been argued that the DU model is unable to explain the preference reversal because it assumes a time independent, constant rate of discounting. In order to explain preference reversal, the model has to have a variable discount rate which changes (decreases) with time. Would decisions made in the Negative Curvature decision space be able to explain such preference reversals?

The discount function is *e*^−*Cosh*^−1^(Γ)^ (from Eq 13). Therefore, the discount rate can be described as

$$\begin{array}{ccc} \psi (m,t^{\prime }) & = & \frac{\frac{-\partial e^{-Cosh^{-1}\left(\Gamma \right)}}{\partial t}}{e^{-Cosh^{-1}\left(\Gamma \right)}}\\ & = & \frac{\eta^{\prime }\left(t\right)}{\Gamma \sqrt{\Gamma^{2}-1}} \end{array}$$ (15)

From Eqs (6) and (8), lim_*t* → ∞_ *ψ*(*m*, *t*′) = 0 i.e., as $t\to \infty ,\frac{\eta^{\prime }\left(t\right)}{\Gamma \sqrt{\Gamma^{2}-1}}=0$. This means that the rate at which the value of reward depreciates in Negative Curvature space is variable and decreases with increasing time. Hence, preference reversals in inter-temporal decisions can be explained by our Negative Curvature model.

### Temporal Sub-additivity

Temporal sub additivity (or sub additive time discounting) is steeper discounting when a delay is divided into parts compared to when it is undivided [40]. For instance, assume the decision-maker is indifferent between $100 now and $150 in 1 year and is also indifferent between $150 in 1 year and $200 in 2 years. However, he appears to prefer $200 in 2 years over $100 now. Therefore, we observe that when the same duration of 2 years is presented as a whole it leads to less discounting as compared to when it is divided into parts. It is inconsistent with both the DU and hyperbolic discounting models. We now show that if we consider the decision space to be negatively curved we can explain temporal sub additivity.

From Eq (10), we know that *d*_*h*_ is a concave function. Therefore, if we keep *m* constant,

$$\begin{array}{c} d_{h}\left(t_{1}+t_{2}\right)\leq d_{h}\left(t_{1}\right)+d_{h}\left(t_{2}\right) \end{array}$$ (16)

In other words, dividing a fixed duration of time into smaller intervals makes it appear more than the same undivided duration.

How would this perception change discounting? Let’s take a duration *t* and divide it into two subdivisions *t*_1_ and *t*_2_ where *t*_1_ + *t*_2_ = *t* while keeping the monetary value constant. Now consider two situations A and B. In A, an initial amount *y*_0_ is first discounted over *t*_1_. If the discounted values at the end of *t*_1_ is *y*_1_ then from Eq (12), $y_{1}=y_{0}e^{-\psi d_{h1}}$ If we further discount *y*_1_ over *t*_2_ then the discounted value at the end of *t*_2_ would be $y_{2}=\left(y_{0}e^{-\psi d_{h1}}\right)e^{-\psi d_{h2}}\Rightarrow \frac{y_{2}}{y_{0}}=e^{-\psi (d_{h1}+d_{h2})}$. On the other hand, lets consider the other situation B where *y*_0_ has been discounted for the entire duration *t*. If *y*_*t*_ is the discounted value at the end of *t*, then $y_{t}=y_{0}e^{-\psi d_{ht}}\Rightarrow \frac{y_{t}}{y_{0}}=e^{-\psi d_{ht}}$. From Eq (16) $d_{ht}<d_{h1}+d_{h2}\Rightarrow \frac{y_{t}}{y_{0}}>\frac{y_{2}}{y_{0}}$. In other words, *y*_0_ will discounted less over the entire time period *t* than when *t* is divided in intervals *t*_1_ and *t*_2_. Hence, our Negative Curvature model can explain temporal sub-additivity.

### Logarithmic time perception

Following the Weber-Fechner and Steven’s law [43], it has been proposed that the perception of time follows logarithmic pattern and the perceived time intervals of the same objective duration gradually shrink as one considers the distant future [14]. Such a shrinkage is a novel notion that is incompatible with both the DU and hyperbolic models. More recently, [15], section 4 also suggested that variable rate discounting may be caused by a non- linear (logarithmic) perception of time by the decision-maker.

However, we can explain such a shrinking in a negatively curved decision space.

Since $Cosh^{-1}\left(\omega \right)=\ln(\omega +\sqrt{\omega^{2}-1})$ where *ω* > 1, using Eq (5)

$$\begin{array}{c} d_{h}=\ln\left(\Gamma +\sqrt{\Gamma^{2}-1}\right) \end{array}$$ (17)

Eq (17) shows that perceived distances in the Negative Curvature decision space follow a logarithmic pattern. Unlike past research which has just shown that time follows a logarithmic pattern, our model predicts such a pattern for both time and money. From Eqs (8) and (9), we can see that the perceived magnitude of an extra unit of time/money in the decision space decreases as the objective value of time/money increases. Therefore, the predictions of Weber-Fechner law or previous logarithmic functions are subsumed in our model.

### Absolute Magnitude Effect

This effect suggests that larger monetary amounts are discounted less steeply than smaller amounts [5, 39]. Consider a decision-maker who is indifferent between receiving $100 now versus $200 after 6 months and is also indifferent between receiving $3000 now versus $4500 in 6 months. This example highlights that for small amounts the discount rate is higher than for large amounts.

We can explain why the absolute magnitude effect occurs if we consider the decision space to be negatively curved. From Eq (15), we see that lim_*m* → ∞_ *ψ*(*m*, *t*′) = 0 which means the discount rate declines as the amount increases. In other words, in the negatively curved decision space for the same duration of time, the discount rate is lower for high monetary outcomes than for low monetary outcomes.

### Preference for Improving Sequences

It has been empirically shown that people prefer improving sequences (where the worst outcome occurs temporally first and the best outcome last) to diminishing sequences (where the best outcome occurs temporally first and the worst outcome last, see [44, 45]).

Again, using a negatively curved decision space we can explain why an improving sequence is preferred. If *m*_3_ > *m*_2_ > *m*_1_ where *m*_1_, *m*_2_, and *m*_3_ are different values of money, we know from Eq (15) that *ψ*(*m*, *t*′) declines with increasing values of *m*. That is, as suggested in explaining the Absolute Magnitude effect, the discount rate decreases with increasing amount of money such that *m*_3_ would depreciate the least and *m*_1_ would depreciate the most. Therefore, if the decision-maker has to temporally order consumption of *m*_1_, *m*_2_, *m*_3_, she would prefer to delay *m*_3_ since it will depreciate the least and consume *m*_1_ the soonest.

Until now, analytically we provided support for our proposal that the inter-temporal decision space resembles the metric properties of Riemannian space of constant negative curvature. We also were able to explain the findings in inter-temporal decision-making using our Negative Curvature model. We next present the second set of evidence where we attempt to learn the Gaussian curvature of the decision space to determine whether it is Euclidean or not.

## Learning the Curvature of the Decision Space

Any attempt to empirically learn the curvature of the decision space poses an intriguing question: if we cannot see the shape of the decision space, how can we infer its curvature. For example, we know that a sphere is not a Euclidean surface because we can observe its shape and find out that it is not a flat surface. However, we don’t have such a vantage point for the inter-temporal decision space, so how can we infer its curvature? The answer to this question lies in Gauss’s “Theorema Egregium” which proves that the Gaussian curvature of a surface, while defined with respect to the higher dimension space that the surface is embedded in, is an intrinsic property of the surface. Our understanding of the earth’s geometry illustrates this very elegantly. Although it has only been a few decades, since we were able to rise above the earth into space and actually observe that the earth is spherical, scientists were able to infer quite accurately from measurements taken on the surface of the earth that its shape was spherical and not flat. More generally, “Theorema Egregium” implies that for any surface a two-dimensional bug living on it, who is unable to holistically view the surface from afar, can still measure the curvature of the surface. Thus, for surfaces with a constant Gaussian curvature, measurements on that surface itself can reveal its nature i.e. whether the surface is elliptical, hyperbolic, or Euclidean. Utilizing this property, we do not need to rise above the decision space or observe it from a distance in order to infer its shape. By taking measurements that inform us about distances on the decision space, we can infer its curvature.

We used Riemannian space learning method to estimate the Gaussian curvature of the inter-temporal decision space. In using this method we face two challenges. First, since the distance estimation process is happening in the mind of the decision maker we cannot visibly see his distance estimates. Second, we cannot provide the decision maker with an objective yardstick for measuring distances i.e. a direct metric assessment of distances. Hence, we use latent distance estimates that are inferred through the inter-temporal choices/tradeoffs the decision-maker makes (we discuss this in detail in later sections).

The Riemannian manifold learning method offers a distinct advantage since we do not need to specify the function that maps the time and money information to the decision space. Similar to any MDS we don’t need to know how objective points are mapped into subjective points. All we need is a measure of distance among each combination of points. Hence, by freeing us from mapping constraints, the procedure provides confirmatory evidence as to the nature of the decision surface. The only input that the method needs in order to determine the nature of the surface is the distance a decision maker perceives between various combinations of money and time. First, we discuss the algorithms to assess the nature of the decision surface (Negative Curvature or Euclidean) then discuss the procedure for collecting the data and how this data was used to infer perceived distances in the decision space.

### Algorithms

We used two different algorithms to test whether the decision surface was Euclidean or Negative Curvature. The input for each algorithm was the $\frac{n(n-1)}{2}$ values of the inter-point distance *d*_*ij*_ (e.g., if *n* = 5, we had ten values of *d*_*ij*_). These algorithms fit values of *d*_*ij*_ to assess if the decision space is Negative Curvature or Euclidean.

For the Negative Curvature algorithm, we utilized Weierstrass coordinates to parametrically represent points on the surface of a hyperboloid [46]. Here (*r*, *θ*) are the polar coordinates in *E*^3^ and *K* is the Gaussian curvature.

$$\begin{array}{c} \left(\begin{array}{c} x_{n}\\ y_{n}\\ z_{n} \end{array}\right)=\left(\begin{array}{c} \frac{1}{\sqrt{K}}Sinh\left[r_{n}\sqrt{K}\right]*Cos\left[\theta_{n}\right]\\ \frac{1}{\sqrt{K}}Sinh\left[r_{n}\sqrt{K}\right]*Sin\left[\theta_{n}\right]\\ \frac{1}{\sqrt{K}}Cosh\left[r_{n}\sqrt{K}\right] \end{array}\right) \end{array}$$ (18)

The Negative Curvature algorithm calculated the following:

1. *n* x 3 values of the Weierstrass coordinates [*x*_*n*_, *y*_*n*_, *z*_*n*_] from *n* x 2 values of the polar coordinates $\left({\hat{\theta }}_{n},{\hat{r}}_{n}\right)$ and the Gaussian Curvature $\hat{K}$.
2. $\frac{n(n-1)}{2}$ values of ${\hat{d}}_{h}\left(i,j\right)$ between all pairs of points *i* and *j* using Weierstrass coordinates from the previous step and Eq (3).
3. $\epsilon^{2}=\sum_{i}\sum_{j}{\left(d_{ij}-{\hat{d}}_{h}\left(i,j\right)\right)}^{2}$

These steps were repeated until *ϵ*^2^ was minimized. In each repetition, the values of $\left({\hat{\theta }}_{n},{\hat{r}}_{n}\right)$ and the Gaussian Curvature $\hat{K}$ were modified using simulated annealing [47]. Once it converged, the Negative Curvature algorithm provided values of the polar coordinates $\left({\hat{\theta }}_{n},{\hat{r}}_{n}\right)$ and $\hat{K}$.

The Euclidean algorithm worked in a similar manner except that the polar coordinates were modified by simulated annealing to approximate the radial distance. The algorithms used here are similar to [48] but with three differences. First, for the Negative Curvature algorithm, we used Weierstrass coordinates to parametrically represent points on the surface, second, we directly measured the Negative Curvature distance (instead of estimating it indirectly from a pseudo Euclidean distance) and third, we used simulated annealing instead of Powell’s method. Please refer to S1 Table for a test of these algorithms with simulated data.

### Procedure

To test our proposed model, we ran two separate studies. The studies used participants who filled out online surveys. The data was anonymous. The study procedure was approved by the University of Utah’s Institutional Review Board. Approval Number: *IRB*_00040903. In study 1, forty participants were recruited from Amazon mechanical turk and completed the study for monetary compensation. In study 2, forty-four undergraduate participants took part in the study for partial course credit. In both studies participants were instructed that they would be making inter-temporal decisions.

Across both studies, a matching procedure was used to elicit participants’ responses to various inter-temporal choices. For instance, participants were asked to fill an amount such that they would be indifferent between the following 2 payoffs: $100 in 1 month versus $___ in 18 months. In the given instance if *y*_*j*_ is the amount filled by the participants, *y*_*i*_ is $100 and *d*_*ij*_ is the perceived distance between *y*_*i*_ and *y*_*j*_ in the decision space, then *d*_*ij*_ can be calculated in the following manner:

We know from Eq 13 that

$$\begin{array}{c} y_{i}=y_{j}e^{-d_{ij}}\\ d_{ij}=ln\left[\frac{y_{j}}{y_{i}}\right] \end{array}$$ (19)

In both studies we used 5 temporal points to elicit different inter-temporal choices (In study 1 these points were: now, 1 year from now, 2 years from now, 5 years from now, and 7 years from now. In study 2 they were: now, 1 month from now, 9 months from now, 18 months from now, and 36 months from now). We also used two different values of *y*_*i*_: $100 and $200. For one set of the matching task we kept *y*_*i*_ as $100 and elicited values of *y*_*j*_ for the five different points in time. We repeated this procedure for another value of *y*_*i*_ as $200. The matching procedure questions with *y*_*i*_ = $100 or $200 were randomized (participants provided a total of 20 values of *y*_*j*_, 10 each for *y*_*i*_ = $100 and *y*_*i*_ = $200). Finally, we calculated the inter-point distance *d*_*ij*_ by averaging $\frac{y_{j}}{y_{i}}$ across *y*_*i*_ = $100 and $200 and using it as the input in Eq (19). The rationale for using two values of *y*_*i*_ was to reduce the impact of outlier responses by using the average value of $\frac{y_{j}}{y_{i}}$.

The data was then used to determine the nature of the decision surface by examining how inter-temporal distances were perceived in the decision space.

### Results

Given the assumptions in past work, our default hypothesis was that the decision space is Euclidean, hence, the base model for our comparison was the Euclidean model. To test whether the Negative Curvature or the Euclidean algorithm provided a better solution, for each participant, we compared their root mean squared errors divided by the standard deviation of the distances. We used the following criteria to exclude participants’ responses from the analysis.

1. In order to test whether participants were paying full attention to the inter-temporal choices, in both studies, we had included a test question, which asked participants to not respond to that question. If participants answered that specific question it would indicate that they were not reading the instructions completely. The program automatically terminated the data collection for such participants.
2. We excluded responses from participants that displayed more than 1 instance of preferring a smaller, later reward over a larger, sooner reward. An example would be someone who is indifferent between receiving $100 today and $200 after 1 year. He is also indifferent between receiving $100 today and $150 after 2 years.

#### Study 1

Of the 40 participants, responses from 5 participants were not used based on the two criteria specified above. Responses from the remaining 35 participants were analyzed by subjecting each participant’s response to both algorithms (detailed results for each participant are summarized in Table A of S2 Table). The Negative Curvature algorithm provided a better fit (lower RMSE/standard deviation ratio) for 25 participants, the Euclidean and the Negative Curvature algorithms provided near identical fit for 6 participants, the Euclidean algorithm provided a better fit for 1, the Negative Curvature fit was less than 1% better than the Euclidean fit for 2 participants, and for 1 participant (participant #29) while the Negative Curvature algorithm provided a better fit, the estimated Gaussian curvature was near zero (indicating a Euclidean solution). Taking a more conservative approach, we classified the cases in which the fit from the Negative Curvature algorithm was less than 1% better than the Euclidean algorithm as a tie and considered all ties as evidence against the negatively curved decision space. This means out of 35 participants, the Negative Curvature algorithm provided a better fit in 25 cases (*χ*^2^(1, *N* = 35) = 6.42, *p* <.01). One could argue that the better fit of the Negative Curvature algorithm is because it has one extra parameter (*K*) than the Euclidean algorithm (where *K* is always zero). The following is one way to address this concern. As shown in Table A in S2 Table, when inter-point distances are sampled from a Euclidean surface, the Negative Curvature algorithm accurately recovers the Gaussian curvature as zero. In other words, this algorithm can identify the Gaussian curvature from both the Euclidean as well as the negative curvature data. If we utilize only the output of this algorithm, we come up with the same conclusion as we arrived at by comparing the Negative Curvature algorithm with the Euclidean algorithm: for 10 participants the Gaussian curvature was near zero while for 25 it was less than zero.

#### Study 2

Of the 44 participants, responses from 6 were not used based on the two criteria discussed above. For the remaining 38 participants, the pattern of result was similar to study 1. The Negative Curvature algorithm provided a better fit (lower RMSE/standard deviation ratio) for 34 participants while the Euclidean algorithm provided a better fit for 3 participants; no difference (or a tie) in fit emerged for the remaining 1 participant. The results are summarized in Table B of S2 Table. Again taking a more conservative approach, we classified the cases in which the fit from the Negative Curvature algorithm was less than 1% better than the Euclidean algorithm as a tie. By doing this, we obtained 31 cases where the Negative Curvature algorithm provided a fit better than 1%, 4 cases where the hyperbolic and the Euclidean algorithm provided largely the same fit (less than 1% improvement) and 3 cases where the Euclidean algorithm provided a better fit. A *χ*^2^ test shows that the Negative Curvature algorithm was a better fit for the data, *χ*^2^(1, *N* = 38) = 15.158, *p* <.0001.

In sum, the results from the two studies provide empirical support to our proposal that the inter-temporal decision space, where the information about time and money is perceived, metrically resembles a Negative Curvature space rather than a Euclidean space.

#### Robustness Check

One criticism of our findings could be that we are assuming a linear transformation of money (i.e., *ϕ*(*y*) = *y*) in Eq (19). We utilized a linear transformation because in much of past research be it animal behavior or human time discounting, researchers have utilized a linear transformation of reward to utility. Therefore, if we consider precedence in terms of empirical testing, the most common functional form was linear. Moreover, it has been found that utilities of small monetary amounts are generally linear [49] and since we were using small amounts such as $100 and $200 we felt that a linear transformation would suffice.

One option to test robustness of our findings is to calculate each participants’ unique *ϕ*(*m*) and then calculate the Gaussian curvature *K*. Abdellaoui et. al [50] discuss a procedure for estimating utility functions in inter-temporal contexts. However, that procedure requires a very different data collection method where time weights are elicited in the first phase and then those weights are used in the temporal discounting task. While this procedure may help us in calculating an individual’s utility function, it may not provide a way to estimate distance *d*_*ij*_ that is needed for our algorithm.

The second option is to utilize an empirically established functional form of *ϕ*(*m*), which happens to be the functional form based on Steven’s law [43] and prospect theory ([51], page 294). So we considered *ϕ*(*m*) = *m*^*α*^. In the gain domain, past work has shown that *α* = .88 and if *α* = 1 then *ϕ*(*m*) = *m* similar to what we assumed in our existing analysis. This resulted in using the equation $d_{ij}=ln\left[\frac{y_{j}^{\alpha }}{y_{i}^{\alpha }}\right]$. Next, to test the robustness of our conclusion that the Negative Curvature space depicts the inter-temporal decision space better than a Euclidean space, we varied alpha from.2 to 1.4 in increments of.2 and used those values to calculate *d*_*ij*_. *d*_*ij*_’s were then used as inputs in both algorithms. The two figures in supporting information S1 Figures depict the results we obtained across both studies (see Figure A in S1 Figures and Figure B in S1 Figures). For all the values of *α*, the data still showed that the Negative Curvature algorithm provided a better fit across studies 1 and 2 compared to the Euclidean algorithm. As expected, since the scale changes (because changing functional forms changes the scaling), we find that the values of *K* change. However, and importantly, the conclusions that a Negative Curvature algorithm provides a better fit to the data remain unchanged.

## Predictions

Analytically and empirically, we have provided evidence to support our proposal that the inter-temporal decision space resembles the metric properties of negative curvature space i.e. it is not Euclidean. As we had mentioned earlier, given that our geometric model of inter-temporal decision making has theoretical underpinnings, not only does it have the ability to explain existing instances of inter-temporal decision making, it also has the ability to make theoretical predictions that can be verified. In this section we put forth some predictions that can be derived from our proposed model.

### Loss/Gain and Gaussian Curvature

Past work has shown that delaying (accelerating) a gain (loss) is discounted more steeply than accelerating (delaying) a gain (loss). The prevailing explanation is rooted in prospect theory, which suggests that since losses loom larger than gains, delaying (accelerating) a gain (loss) is an aversive outcome and thus, felt more acutely than accelerating (delaying) a gain (loss).

Using our geometric model, if *ψ*(*m*, *t*′) is the discount rate then the loss/gain discounting asymmetry can be expressed as *ψ*(*m*, *t*′)_*loss*_ > *ψ*(*m*, *t*′)_*gain*_. If in Eqs (14) and (15), we substitute *K*_*L*_ and *K*_*G*_ as the Gaussian curvatures of the decision space in the loss and gain domain respectively, then we can depict the loss/gain discounting asymmetry in the Negative Curvature decision space as

$$\begin{array}{c} \begin{array}{c} \frac{-\frac{\partial e^{-\frac{Cosh^{-1}\left(\sqrt{-K_{L}}\sqrt{\phi^{2}\left(m\right)+\eta^{2}\left(t\right)-\frac{1}{K_{L}}}\right)}{\sqrt{-K_{L}}}}}{\partial t}}{e^{-\frac{Cosh^{-1}\left(\sqrt{-K_{L}}\sqrt{\phi^{2}\left(m\right)+\eta^{2}\left(t\right)-\frac{1}{K_{L}}}\right)}{\sqrt{-K_{L}}}}}>\frac{-\frac{\partial e^{-\frac{Cosh^{-1}\left(\sqrt{-K_{G}}\sqrt{\phi^{2}\left(m\right)+\eta^{2}\left(t\right)-\frac{1}{K_{G}}}\right)}{\sqrt{-K_{G}}}}}{\partial t}}{e^{-\frac{Cosh^{-1}\left(\sqrt{-K_{G}}\sqrt{\phi^{2}\left(m\right)+\eta^{2}\left(t\right)-\frac{1}{K_{G}}}\right)}{\sqrt{-K_{G}}}}} \end{array} \end{array}$$ (20)

This inequality would hold only if *K*_*G*_ < *K*_*L*_ where both *K*_*G*_ and *K*_*L*_ are less than zero. Please see S4 Text for the complete solution.

It means for delayed-gains to be discounted more than accelerated-gains, the Gaussian curvature of the decision space has to be less when a person considers a delayed-gain than when he considers an accelerated-gain (or an accelerated-loss than a delayed-loss).

Predicting that the Gaussian curvatures for gains and losses would be different is an intriguing conjecture because it is analogous to the description of a malleable geometry suggested by Riemann that we had discussed earlier. It would mean that the decision space is not a rigid container with a fixed geometry; instead its geometry (based on different curvatures) evolves with changes in the decision context. It leads to the proposal that inter-temporal decisions in the loss and gain domain are different because loss and gain differentially alter the curvature of the decision space.

We can extend this to make some interesting predictions in other domains where dispositional or visceral factors are known to influence the discount rate. For instance, [52] showed that children are able to display higher self-control with symbolic versus real reward (e.g., more likely to be tempted by a real cake than an image of it). Similarly, [39], page 595 discuss how proximity to sensory contact with a choice object increases the discount rate. Our model would predict that proximity to sensory rewards influences the discount rate by altering the curvature (and hence, the geometry) of the decision space.

### Monetary subadditivity

In our geometric model, we can see that Eq (5) is sub-additive not just for time but also for money. In other words, for a constant time duration

$$\begin{array}{c} d_{h}\left(m_{1}+m_{2}\right)\leq d_{h}\left(m_{1}\right)+d_{h}\left(m_{2}\right) \end{array}$$ (21)

Eq (21) would predict that the same amount of money over a fixed duration will be discounted more when divided in parts than as a whole. In other words, if $*x*1 + $*x*2 = $*X* then $*X* will be discounted less than the aggregate of $*x*1 and $*x*2 over a fixed time duration *t*. For example, our model would predict that the discounted value of $500 in 1 year will be more than the sum of discounted values of $200 and $300 in 1 year.

### Subadditivity at distant time periods

As we discussed earlier, inter-temporal subadditivity is inconsistent with both the DU and hyperbolic discounting models, but it can be explained if we conceptualize the decision space to be negatively curved. However, very little is known about inter-temporal subadditivity at distant time periods. In other words, for the distant-future, would we observe a similar subadditive influence by dividing the time duration into smaller intervals as we observe for proximate time or would temporal subadditivity attenuate at distant times?

We know from Eq (16) that if we divide a time duration *t* into $\frac{t}{n}$ and $\frac{(n-1)t}{n}$ then

$$\begin{array}{c} d_{h}\left(\frac{t}{n}+\frac{(n-1)t}{n}\right)\leq d_{h}\left(\frac{t}{n}\right)+d_{h}\left(\frac{(n-1)t}{n}\right),\forall n>0 \end{array}$$

If we add interval *g* to *t*

$$\begin{array}{c} \Rightarrow d_{h}\left(\frac{\left(t+g\right)}{n}+\frac{\left(n-1)(t+g\right)}{n}\right)\leq d_{h}\left(\frac{\left(t+g\right)}{n}\right)+d_{h}\left(\frac{\left(n-1)(t+g\right)}{n}\right),\forall n>0,g\geq 0 \end{array}$$

$$\begin{array}{c} \Rightarrow \frac{\partial d_{h}\left(\frac{\left(t+g\right)}{n}+\frac{\left(n-1)(t+g\right)}{n}\right)}{\partial t}\leq \frac{\partial d_{h}\left(\frac{\left(t+g\right)}{n}\right)}{\partial t}+\frac{\partial d_{h}\left(\frac{\left(n-1)(t+g\right)}{n}\right)}{\partial t} \end{array}$$

Now if *g* → 0 then

$$\begin{array}{c} \underset{g\to 0}{\text{lim}}\left[\frac{\partial d_{h}\left(\frac{\left(t+g\right)}{n}+\frac{\left(n-1)(t+g\right)}{n}\right)}{\partial t}\right]\leq \underset{g\to 0}{\text{lim}}\left[\frac{\partial d_{h}\left(\frac{\left(t+g\right)}{n}\right)}{\partial t}+\frac{\partial d_{h}\left(\frac{\left(n-1)(t+g\right)}{n}\right)}{\partial t}\right] \end{array}$$ (22)

Consistent with existing research, Eq (22) shows that in the near-future discounting will be more if the time duration is divided into intervals compared to when the same duration is kept undivided.

However, if *g* → ∞ then

$$\begin{array}{c} \underset{g\to \infty }{\text{lim}}\left[\frac{\partial d_{h}\left(\frac{\left(t+g\right)}{n}+\frac{\left(n-1\right)\left(t+g\right)}{n}\right)}{\partial t}\right]∽\underset{g\to \infty }{\text{lim}}\left[\frac{\partial d_{h}\left(\frac{\left(t+g\right)}{n}\right)}{\partial t}+\frac{\partial d_{h}\left(\frac{\left(n-1\right)\left(t+g\right)}{n}\right)}{\partial t}\right] \end{array}$$ (23)

Therefore, Eq (23) predicts that at distant time periods, the subadditive influence on discounting would be attenuated.

## Conclusions

In this research we question the geometry underlying the inter-temporal decision space and build a theoretical model based on an appropriate geometry that better explains the existing empirical findings (anomalous or not) and helps make further theoretical predictions. Past research has assumed that the decision space (where time and money are perceived/experienced by the decision-maker) is an Euclidean space. However, we propose that the decision space is a Riemannian space with constant Negative Curvature.

We support our proposition through two approaches. First, we provide evidence, analytically, by deriving a new discount function, which uses the distance in the Negative Curvature space instead of Euclidean temporal distance. By doing so we are able to explain the empirical anomalies that have been shown in the inter-temporal literature. In other words, empirical anomalies (such as common difference effect, absolute magnitude effect, temporal subadditivity, logarithmic time perception, preference for improving sequence) that at times are inconsistent with the DU model and/or the hyperbolic discounting model can be explained by considering the geometry of the decision space to be non-Euclidean. Second, when we measure the Gaussian curvature of the decision space through surface learning algorithms we find that the metric properties of the decision space resemble those of the Negative Curvature space rather than the Euclidean space.

By building a geometric model of the inter-temporal decision space, we question a widely accepted notion that the surface underlying the decision space is Euclidean. By relaxing this rigid assumption, we propose that a more flexible approach should be adopted so that we can take into account dispositional factors of the decision-maker, as well as contextual factors in which the decision is being made, to influence the geometry of the decision space and thereby the metric relationship among the decision objects. Finally, by considering both time and money in the distance function we are suggesting that both, together, influence inter-temporal decisions. Such an integration of money helps us get richer insights rather than when only time is considered to be the sole influencer.

If we look at the discount function in Eq (12), we find that it is analogous to the DU discount function with one difference. Instead of assuming the inter-temporal space (where discounting happens) to be a Euclidean space, we are assuming it be a more flexible negatively curved space. One intriguing outcome of relaxing the rigid Euclidean assumption is that the DU model, much criticized for its inability to explain anomalies, can now explain many anomalies. It also raises questions about how we define non-normative behavior and anomalies. By assuming the wrong geometry we may conclude that the decision maker is behaving non-normatively. However, our conclusion is flawed as it is based on the wrong assumption. Let’s consider the analogy of a bug moving on a transparent globe to illustrate this point. Assume that the bug is traveling along the great circle, which is the shortest (geodesic) path on a globe. However, assume further that we cannot observe the bug’s actual movements. All we can see is the shadow of the bug’s movement, including the start and end points, on the floor caused by a light bulb kept at the top of the globe. We are unaware of the shape of the object on which the bug is actually moving, whether it is a globe, a cylinder, a saddle or a flat surface. Since, the only thing we observe is the shadow on the floor, we could erroneously assume that the bug is moving on a flat surface and try to predict the shortest distance it should move to go from the start to the end point. By this error in our assumption, we would find the bug’s path to be quite irrational (at times following a straight line when moving along the prime meridian on the globe and at times a curved path as we see its shadow moving along the equator) since it would not be moving by the shortest distance predicted by a flat surface. Unfortunately, it will be our conclusion that is wrong since we are presuming the movement on the wrong surface; the bug is quite consistent in its movement as it follows the shortest path on the globe, which is the great circle.

Similarly, if we replace the globe with the decision space and the bug’s path with how humans estimate inter-temporal distances, we see that it is our assumption of the geometry of the space to be Euclidean which is at fault, rather than the decision-maker’s inter-temporal choices. If we erroneously use the Euclidean distance between two inter-temporal points to estimate how much an outcome needs to be discounted, we would reach the incorrect conclusion that the decision-maker is non-normative. However, in reality the decision makers are correctly estimating the distance along the shortest path but their decision space is negatively curved. Therefore, if we identify the right geometry underlying their decision space, we would see that they are actually responding normatively. In sum, a more flexible approach is to recognize that the decision objects don’t fit into some pre-specified Euclidean space (with its established geometric metric) but that the decision space is defined by the factors present during the decision making process and can be more malleable than the rigid Euclidean space.

## Supporting Information

S1 Table Testing the Algorithms. (TEX)

S2 Table Results. Table A, study 1 results. Table B, study 2 results.
(TEX)

S1 Figures Robustness checks. Figure A, Study 1 (Euclidean vs. Negative Curvature Algorithm fit). Figure B, Study 2 (Euclidean vs. Negative Curvature Algorithm fit).
(TEX)

S1 Text Symbols. (TEX)

S2 Text Riemannian Space. Text A, Gaussian Curvature. Text B, Constant Negative Curvature.
(TEX)

S3 Text Mathematical details. Text A, Derivation of the distance equation for the hyperboloid model (Eqs 2 and 3). Text B, Deriving the distance equation for the inter-temporal decision space (Eqs 4 and 5). Text C, Verification of Eq 5. Text D, Solution of Eq 11.
(TEX)

S4 Text Prediction in the loss and gain domain. (TEX)
